# A Proteomics Resource Investigating Fibrosis: Proof‐of‐Concept for Identifying Novel Drug Candidates

**DOI:** 10.1002/pmic.70090

**Published:** 2025-12-12

**Authors:** Hanne Devos, Manousos Makridakis, Rafael Stroggilos, Mayra Alejandra Jaimes Campos, Aggeliki Tserga, Marika Mokou, Maria G. Roubelakis, Jerome Zoidakis, Antonia Vlahou, Agnieszka Latosinska

**Affiliations:** ^1^ Centre of Systems Biology Biomedical Research Foundation of the Academy of Athens Athens Greece; ^2^ Mosaiques Diagnostics GmbH Hannover Germany; ^3^ Laboratory of Biology National and Kapodistrian University of Athens, School of Medicine Athens Greece; ^4^ Laboratory of Cell and Gene Therapy Biomedical Research Foundation, Academy of Athens Athens Greece

**Keywords:** common fibrotic signature, drug repurposing, extracellular matrix, fibrosis, publicly available proteomics data

## Abstract

Fibrosis is characterised by inappropriate wound healing and the buildup of excessive fibrous connective tissue, in particular within the extracellular matrix (ECM). This can occur in multiple organs, ultimately leading to organ failure. Despite the high burden of fibrosis, treatment options only delay disease progression. Therefore, leveraging publicly available proteomics data, we investigated whether common fibrotic proteins and pathways in different organs could be found, to define potential core changes related to fibrosis. We identified 124 significantly differentially expressed proteins in heart fibrosis, four in early‐versus‐mild liver fibrosis, 135 in mild‐versus‐severe liver fibrosis and 160 in early‐versus‐severe liver fibrosis. Functional annotation of each of these groups of proteins demonstrated a consistent upregulation of ECM proteins and a consistent downregulation of proteins associated with mitochondrial activity. Using these data for drug repurposing, 26 compounds were proposed for further investigation, with 20 of them having demonstrated a promising anti‐fibrotic effect. A core set of 18 proteins were shared between heart and liver fibrosis, and are associated with increased ECM deposition and fibroblast activation. This approach can be generalised for other pathologies, improving the knowledge on the affected molecular pathways, and based on this, identifying potential drug candidates/compounds.

**Abbreviations**
BH: Benjamini–Hochberg correction for multiple testing
DDA: data‐dependent acquisition
DIA: data‐independent acquisition
ECM: extracellular matrix
EMT: epithelial‐mesenchymal transition
GSVA: gene set variation analysis
Nedrex: Network‐based Drug Repurposing and exploration

1

Fibrosis is characterised by inappropriate wound healing and can manifest in multiple organs (such as liver, heart, kidney, lung), due to various tissue injuries [1]. Despite organ‐specific differences, commonly in all affected organs, increased inflammation and deposition of extracellular matrix (ECM) proteins are observed, eventually leading to organ failure [1]. The most frequently affected organs are liver (affecting one in four persons of the global population), kidney (affecting one in six), heart (affecting one in 60) and lung (affecting one in 1500) [2]. Despite the high burden of fibrosis [2], clinical treatment only delays disease progression [2], although recent progress has been made in the field of liver fibrosis [3]. Therefore, there is a high unmet need to better understand fibrotic disease mechanisms, driving evidence‐based shortlisting of anti‐fibrotic drugs [4]. To facilitate investigation of fibrosis at the molecular level, we established a proteomics resource compiling existing relevant datasets retrieved following a systematic search. Using this resource, we investigated whether a common fibrotic protein signature which shared differentially regulated pathways in different organs (heart, liver) could be identified. Finally, we performed molecularly–driven drug repurposing, with further in silico verification of the biological relevance of the identified drug candidates. This approach can be generalized to different diseases, contributing to a better understanding of disease‐associated molecular changes and leveraging that knowledge to propose drug candidates.

Publicly available proteomics datasets from ProteomeXchange and its repositories PRIDE and MassIVE [5] were identified, focussing on chronic diseases associated with fibrosis in heart, liver or kidney. More details on inclusion criteria and employed keywords are provided in Supporting Information. In total, three datasets on heart (heart fibrosis dataset 1 (PXD008934), heart fibrosis dataset 2 (PXD012467) [7], heart fibrosis dataset 3 (PXD054266) [8]), three datasets on liver (liver fibrosis dataset 1 (PXD001474) [9], liver fibrosis dataset 2 (PXD027722) [10] and liver fibrosis dataset 3 (MSV000094959) [11]) and two datasets on kidney fibrosis (PXD006339 [12] and PXD040617 [13]) were selected (Table S1). Briefly, data were re‐analysed using Proteome Discoverer v1.4 for Data‐Dependent Acquisition (DDA) or DIA‐NN v1.9.1 for data‐independent acquisition (DIA) data, as applicable. For datasets passing quality control, the protein exports are available in Table S2. For the heart and liver datasets, after removal of samples and proteins with more than 70% missing values, Wilcoxon Rank Sum tests were used to identify differential protein expression, followed by Benjamini–Hochberg (BH) correction for multiple testing. The two kidney datasets were not considered for further analysis due to quality concerns (mainly blood contamination, details in Supporting Information). For the remaining datasets, proteins with a BH‐adjusted *p* value < 0.05 and nominal significance in at least one other dataset examining the same organ, as well as a consistent trend in log_2_ fold change across all datasets for that organ, were considered significant. STRING network analysis [14, 15] and Gene Set Variation Analysis (GSVA) [16] using MSigDB [17] on differential expressed proteins were further applied for pathway mapping. Finally, using Network‐based Drug Repurposing and exploration (NedRex) [4] and Cytoscape version 3.10.3 [18] drug repurposing analysis was performed to shortlist interesting compounds targeting key proteins and pathways in fibrosis, independent of aetiology.

In heart fibrosis, 124 proteins were shared among at least two out of three datasets (significant after BH correction in at least one dataset, nominally significant in at least one other dataset, with a concordant trend in log_2_ fold change across all heart datasets) (Table S3). Similarly, four shared proteins were identified in early‐versus‐mild liver fibrosis (Table S4), 135 in mild‐versus‐severe liver fibrosis (Table S5) and 160 in early‐versus‐severe liver fibrosis (Table S6). In Tables S3–S6, the ppm normalised and log_2_‐transformed average abundance values of cases and controls, as well as respective standard deviation, log_2_ fold change, and (un)corrected *p* values are reported per protein and dataset. The low number of shared proteins in early‐versus‐mild liver fibrosis was reported earlier [19]. Moreover, it should be noted that all four proteins in the early‐versus‐mild comparison were significant after BH correction in liver fibrosis dataset 2 (PXD027722) and nominally significant in liver fibrosis dataset 3 (MSV000094959). Eighteen proteins were common between heart fibrosis and liver fibrosis and demonstrated concordant upregulation in fibrosis versus non‐/early fibrosis stages, and are mostly ECM‐related (*n* = 14) (based on matrisomeDB annotation [20]) (Table S7, also providing the results of the statistical analysis of each of the individual datasets), and are listed along with their role in heart and liver fibrosis in Table B (Supporting Information), and graphically shown in Figure 1.

**FIGURE 1:**
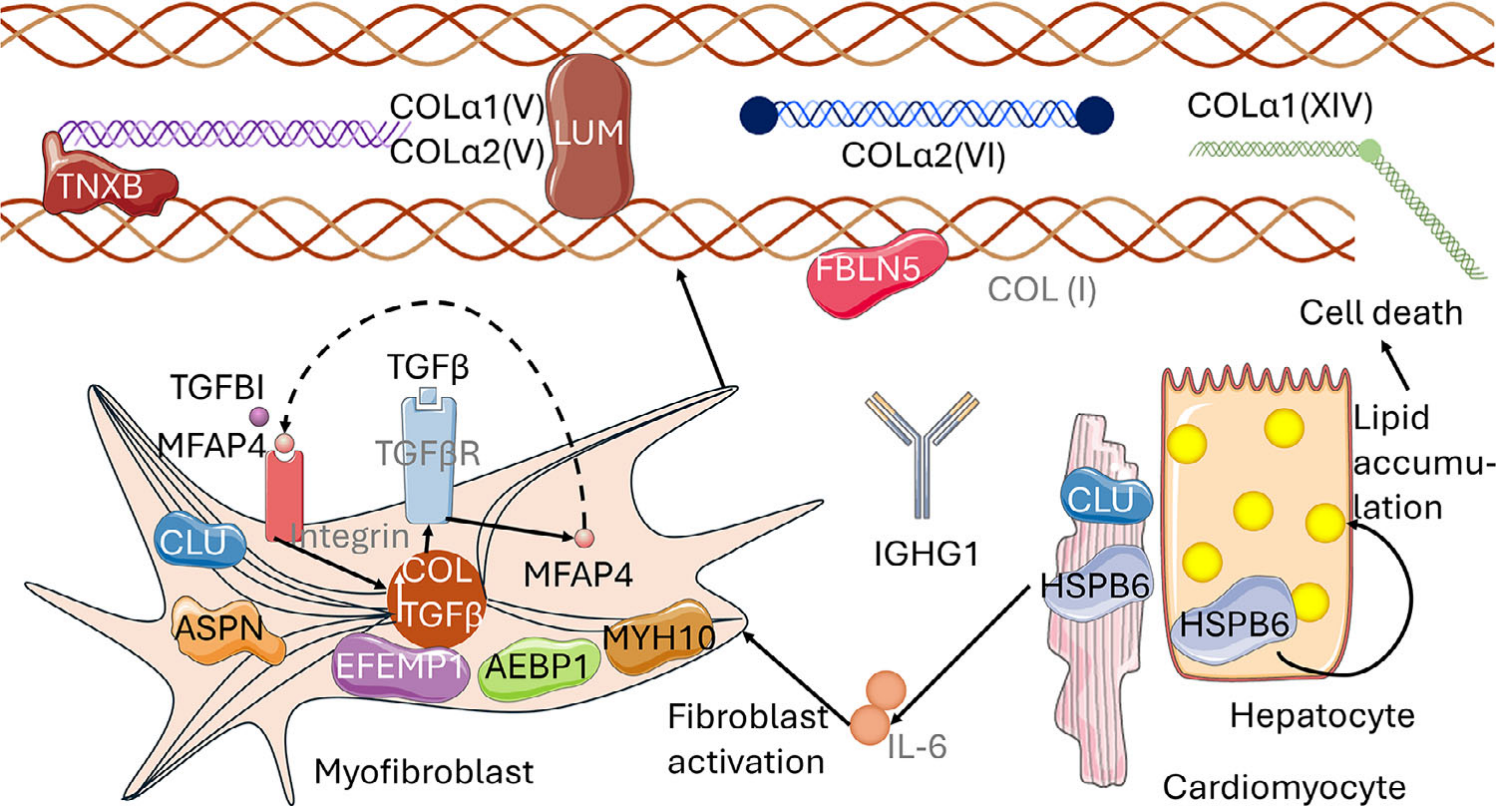
Overview of the role of the shortlisted proteins in heart and liver fibrosis. Full protein names are shown in Table S7, along with the gene names shown in this figure. Proteins with names shown in grey were not detected and are included solely to illustrate the function of black‐ and white‐labelled proteins.

Using the protein‐protein interaction database StringDB [14, 15], an in silico analysis of interactions among the shared differential expressed proteins in heart fibrosis and subsequently across the three liver fibrosis comparisons was performed. Considering the low number of shared, concordant protein changes in early‐versus‐mild liver fibrosis (*n *= 4) as above mentioned, no network could be generated. The three remaining networks (generated using the 124 heart fibrosis proteins, 135 mild‐vs.‐severe liver fibrosis proteins and 160 early‐vs.‐severe liver fibrosis proteins), shown in Figures S7–S9, were clustered using Markov Clustering Algorithm [14, 15] and exhibited an upregulation of proteins associated with the ECM and a downregulation of proteins involved in cell metabolism (Figures S10 and S11).

GSVA [16] analysis, as described in the Supporting Information, further supported that shared (common) pathways between heart fibrosis and liver fibrosis reflected reduced mitochondrial activity and catabolic processes, alongside increased ECM synthesis (specifically collagens and proteoglycans) and degradation, cell‐matrix interactions, response to transforming growth factor β‐related signalling, and epithelial‐mesenchymal transition (EMT) in agreement with earlier findings [2, 21]. The fifteen most significant (de‐)activated pathways shared between heart fibrosis and mild‐versus‐severe liver fibrosis, and heart fibrosis and early‐versus‐severe liver fibrosis are shown in Figure S12, and the full list is available in Tables S8 and S9, respectively.

Finally, NedRex [4] was employed, using as input the 124 heart fibrosis proteins, or the 135 proteins from the mild‐versus‐severe liver fibrosis or the 160 proteins from the early‐versus‐severe liver fibrosis comparisons. Compiling the results from these three analyses, 26 compounds were identified as potential therapeutic candidates based on the heart fibrosis proteins and the proteins of either of the liver fibrosis comparisons (Figure 2, Table S10). The targets of these drugs are shown in Figure 2 (in case of direct interaction with our shortlisted proteins) or listed in Table S10 (for all drugs, along with an indication whether the interaction is direct or indirect). For 20 of them, pre‐clinical research or an early clinical trial supports their relevance in both heart and liver fibrosis (Zinc [22, 23], early‐clinical trial [24]; Quercetin [25, 26]; Imatinib [27, 28]; Nintedanib [29, 30], approved for idiopathic pulmonary fibrosis [31]; Dasatinib [32, 33]; Sorafenib [34, 35]; Axitinib [36, 37]; Erlotinib [38, 39]; Tamoxifen [40, 41]); heart fibrosis (Pyridoxal phosphate [42]; Ruboxistaurin [43]; Miconazole [44]; Copper [45]), or liver fibrosis (Sunitinib [46, 47]; Nilotinib [48]; Ruxolitinib [49] and approved for myelofibrosis [50]; Neratinib [51]; Pazopanib [52]; Gefitinib [53]; Chlorpromazine [54]). Nicotinamide adenine dinucleotide hydrogen (NADH) [55, 56] was predicted yet with no reported role in fibrosis. The network depicting the protein‐compound interactions is shown in Figure 2.

**FIGURE 2:**
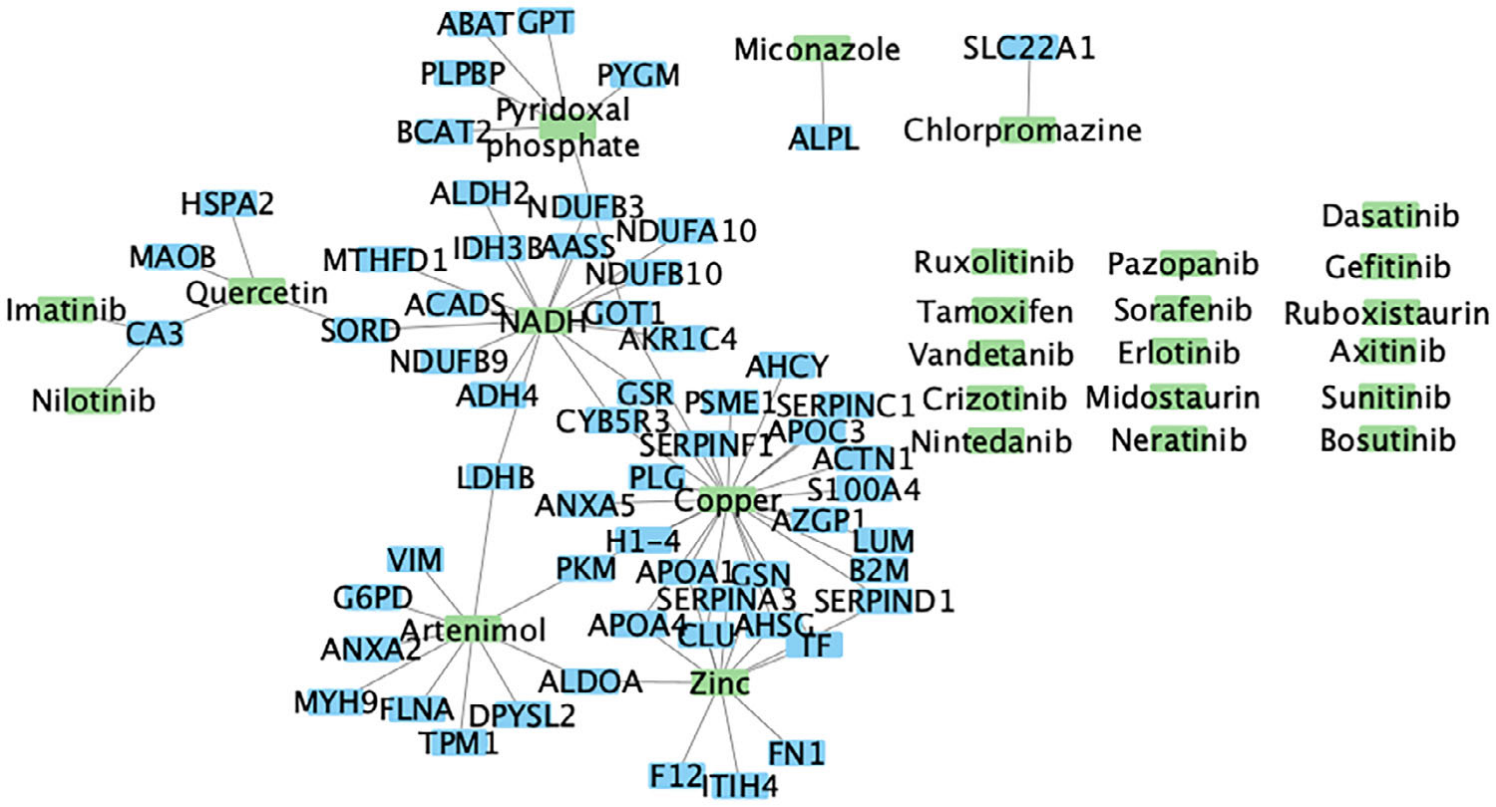
Protein–Protein and Compound–Protein interaction network. Green indicates drug candidates or compounds relevant to fibrosis; blue the proteins they target. For some, no direct target was identified; instead, they were identified through indirect targets (interacting with the shortlisted proteins) (not shown) (Table S10).

Interestingly, 17 out of 26 compounds are protein kinase inhibitors (Table S10). Indeed, as shown in previous studies, kinases play a vital role in fibrosis [57, 58, 59], and represent an attractive therapeutic target [60]. Nevertheless, challenges remain, such as off‐target effects which lead to toxicity [60]. Other post‐translational modifications have also been reported to play a significant role in heart [61] and liver fibrosis [62] (e.g., oxidation, acetylation, glycation, succinylation and S‐palmitoylation [61, 62]), and efforts are ongoing to explore their drugability [62].

These results highlighted that despite tissue‐specific differences, common disease processes between heart and liver fibrosis may exist. Collectively, using publicly available proteomics data, 18 shared differential expressed proteins in heart and liver fibrosis were identified, reflecting fibroblast activation and ECM remodelling. It should be noted, that in the liver fibrosis analysis in general most proteins were significant after BH correction in liver fibrosis dataset 2 (PXD027722), which may be attributable to a more even distribution of patients across the respective subgroups in this dataset. In addition, it is important to note that due to the heterogeneity in the number of identified proteins between datasets, unintentional bias may have been introduced. Moreover, our significance criterion of not requiring adjusted *p* values to be significant in all datasets may unintentionally have identified false positives. Nevertheless, we have required presence in multiple datasets to reduce bias and have carefully validated our findings in literature which collectively minimizes the risk of false positives. In addition, this is to our knowledge the first resource presenting shared molecular features of fibrosis in different organs.

This resource can be reliably used to improve the understanding of heart and liver fibrosis and whether they have shared molecular features, as well as aid in the prioritisation of further investigation of relevant drugs. The employed pipeline may be generalized and applied in the context of other diseases.

## Conflicts of Interest

Mayra Alejandra Jaimes Campos, Marika Mokou, and Agnieszka Latosinska are employed by Mosaiques Diagnostics GmbH. Hanne Devos is now employed by Nordic Bioscience A/S. The remaining authors declare no conflict of interest.

## Supporting information




**Supporting file 1**: pmic70090‐sup‐0001‐SuppMat.zip

**Supporting file 2**: pmic70090‐sup‐0002‐Tables.zip

## Data Availability

Associated data has been deposited on ProteomeXchange (heart fibrosis dataset 1 (PXD008934) [6], heart fibrosis dataset 2 (PXD012467) [7], heart fibrosis dataset 3 (PXD054266) [8], liver fibrosis dataset 1 (PXD001474) [9], liver fibrosis dataset 2 (PXD027722) [10], PXD006339 [12] and PXD040617 [13]) or MassIVE (liver fibrosis dataset 3 (MSV000094959) [11]). Protein identification and abundance data is available in Table S2.
