# Supplementary material for: A Proteomics Resource Investigating Fibrosis: Proof‐of‐Concept for Identifying Novel Drug Candidates
Source: Proteomics. 2025 Dec 12;26(1):94–9. doi: 10.1002/pmic.70090 (PMC12809004; doi:10.1002/pmic.70090)
Supplement: Supplementary file 1 — Supporting file 1: pmic70090‐sup‐0001‐SuppMat.zip [file PMIC-26--s001.docx]

**Supplementary Materials**

**Search of relevant datasets**

A comprehensive search in the proteomics datasets from ProteomeXchange and its repositories PRIDE and MassIVE [1] was conducted to identify relevant datasets. The following keywords were used: “heart”, “cardiomyo”, “hypertension”, “obesity”, “cardiac” to identify heart fibrosis-related datasets; “liver”, “non-alcoholic”, “metabolic dysfunction”, “non-alcoholic fatty liver disease”, “non-alcoholic steatohepatitis”, “obesity”, “hepatic” for liver fibrosis-related datasets; and “kidney”, “chronic kidney disease”, “type 1 diabetes mellitus”, “type 2 diabetes mellitus”, “diabetic nephropathy”, “end-stage renal disease”, “renal” for kidney fibrosis-related datasets. In addition, the keyword “fibrosis” was used, manually screening for datasets on heart, kidney or liver tissue. The keywords were used subsequently in each repository, and the search was completed in September 2024. A total of 509 datasets for heart, 581 datasets for liver, and 514 datasets for kidney were retrieved. An overview of the number of excluded datasets per exclusion criterion can be found in the **Table A** below, and exclusion criteria were applied in the order they are listed. Following each search, datasets were manually evaluated for inclusion, with the following exclusion criteria: cell lines, as their changes may not fully reflect changes in native tissue and differences in culturing conditions and stimuli would further complicate the analysis; commercial samples; diseases not associated with fibrosis (explained in more detail below); non-tissue proteomics (mostly blood and urine), as we aimed to map changes on the tissue level; datasets where the disease status was unclear (mostly in the context of method optimisation or generation of tissue proteome maps); non-adult samples, as the tissue proteome is likely to change with age; use of labelling, enrichment strategies, or targeted proteomics, to maintain an unbiased view of the changes in the fibrotic tissue proteome; datasets with N<10, due to the lack of statistical power; datasets only studying healthy tissue, as they were incompatible with the followed workflow described below in the section ‘statistical analysis’; other types of (prote-)omics, including chemoproteomics, ChIP-MS, cross-linking mass spectrometry, degradomics, immunopeptidomics, lipidomics, metabolomics, middle-down proteomics, paleoproteomics, peptidomics, spatial proteomics, top-down proteomics and toxicoproteomics, due to the different type of results obtained and different data analysis strategies; datasets for which no publication could be found, despite google search of the dataset accession number and title, as the publication was needed for comparison of the initial results. For heart, only samples from the left ventricle were considered, as changes in the left ventricle are more likely to reflect chronic heart fibrosis. The following diseases were excluded: amyloidosis (characterised by the deposition of misfolded proteins [2], whereas fibrosis is characterised by chronic inflammation [3]), long COVID, as a link to chronic inflammation has not been proven yet [4], acute rejection after transplantation (as this reflects acute inflammation rather than chronic inflammation), cancer (as the tumour in itself induces changes to the tissue proteome, which will be difficult to distinguish from the fibrosis-induced changes) [5], atrial fibrillation (characterised by fibrosis in the atrial walls, while we focussed on the left ventricle [6]).

Table A: Overview of the number of datasets excluded. Each reason is explained in more detail in the text above.

| Exclusion reason | Heart | Liver | Kidney |
| --- | --- | --- | --- |
| Duplicate datasets (found by more than one search term) | 158 | 106 | 59 |
| Datasets using only cells (instead of primary tissue) | 147 | 166 | 252 |
| Datasets not focussing on solid tissue (blood/urine proteomics) | 36 | 59 | 58 |
| Disease not associated with fibrosis induced by chronic diseases (in more detail discussed above) | 23 | 105 | 61 |
| Disease status unclear (tissue maps, method optimisation) | 15 | 30 | 16 |
| Paediatric samples | 7 | 6 | 5 |
| Commercial samples | 0 | 1 | 0 |
| Not from left ventricle | 1 | 0 | 0 |
| Use of labelling strategy (iTRAQ, TMT) | 17 | 27 | 18 |
| Enrichment strategies (post-translational modifications, interactomics for specific proteins) | 46 | 30 | 14 |
| Targeted proteomics | 7 | 11 | 4 |
| Low sample size (n<10) | 2 | 7 | 0 |
| Only healthy tissue included | 3 | 4 | 7 |
| No healthy controls (only disease versus other disease) | 3 | 0 | 0 |
| Other types of (prote)-omics | 20 | 25 | 15 |
| No publication (even after google search of the dataset) | 8 | 1 | 2 |

Finally, three datasets were retained on heart fibrosis, as well as three datasets on liver fibrosis, and two datasets on kidney fibrosis.

**Peptide filtering and summary to protein level**

Datasets obtained using data-dependent acquisition (DDA) were re-analysed using Proteome Discoverer 1.4. Datasets obtained using data-independent acquisition (DIA) were re-analysed using DIA-NN v1.9.1. In both cases, the human proteome database obtained from UniProtKB (version May 2024, including reviewed, canonical sequences) in FASTA format was used for the analysis. The following search settings were specified: cleaving agent: trypsin; number of missed cleavages: 2; precursor mass tolerance: 5 ppm; fragment mass tolerance: 0.05 Da (Proteome Discoverer) or 5 ppm (DIA-NN); modifications: carbamidomethylation of cysteine (fixed), oxidation of methionine (variable), oxidation of proline (variable). Specifically for DIA-NN, the precursor and fragment m/z range were set to 250-1650, peptide length was set to be between 7-100 amino acids, and precursor charge range was set at 1-4.

Following the search, the peptides were exported and further filtered as follows: proline hydroxylation was only permitted on collagen-derived peptides and peptides carrying an oxidized methionine were only retained if a peptide with the same amino acid sequence but without that oxidized methionine was also identified. Abundances of peptides assigned to each protein were then summed to obtain protein abundances.

**Initial quality control of data**

As a first step, the number of identified proteins per dataset was compared to the respective publication, allowing discrepancies between 25% and 175% of the originally reported number of proteins identified in the original publication. Two datasets failed this initial quality control and were further inspected. In case of PXD006339 (kidney fibrosis), in which less than 100 proteins were identified per sample, none of which could be quantified, manual inspection of the dataset raised severe concerns (peptide separation was poor, low overall intensity and poor quality of the total ion chromatogram was observed, whereas peptide abundance could not be estimated by Proteome Discoverer), which led to the exclusion of this dataset. In case of PXD040617 (kidney fibrosis), a more than 250% increase of identified proteins (as compared to the number of proteins identified mentioned in the original publication) was observed, outside of the 25%-175% discrepancy limit mentioned above, as well as highly abundant blood-derived proteins, potentially indicating blood contamination. For these reasons, this dataset was also removed from the analysis.

For the three remaining datasets on heart fibrosis and liver fibrosis respectively, quality control was continued by removing samples with more than 70% missing values and/or 0 values. This led to the removal of one heart failure sample in heart fibrosis dataset 2 (PXD012467); no samples were removed in the liver failure datasets. Moreover, six compensated hypertrophy samples from heart fibrosis dataset 1 (PXD008934) were not considered, as this disease is not generally associated with fibrosis. Proteins with more than 70% missing values and/or 0 values were also removed. The original number of identified proteins and relevant samples, as well as the number of proteins and samples retained following this filtering step is noted in **Table S1**. The number of identified proteins after filtering as described above, in each comparison, for each dataset, is shown in **Figure S1**. While there are differences in the number of identified proteins in the datasets, they are mostly related to advances in instruments used and align with the reported number of identified proteins in the original publications.


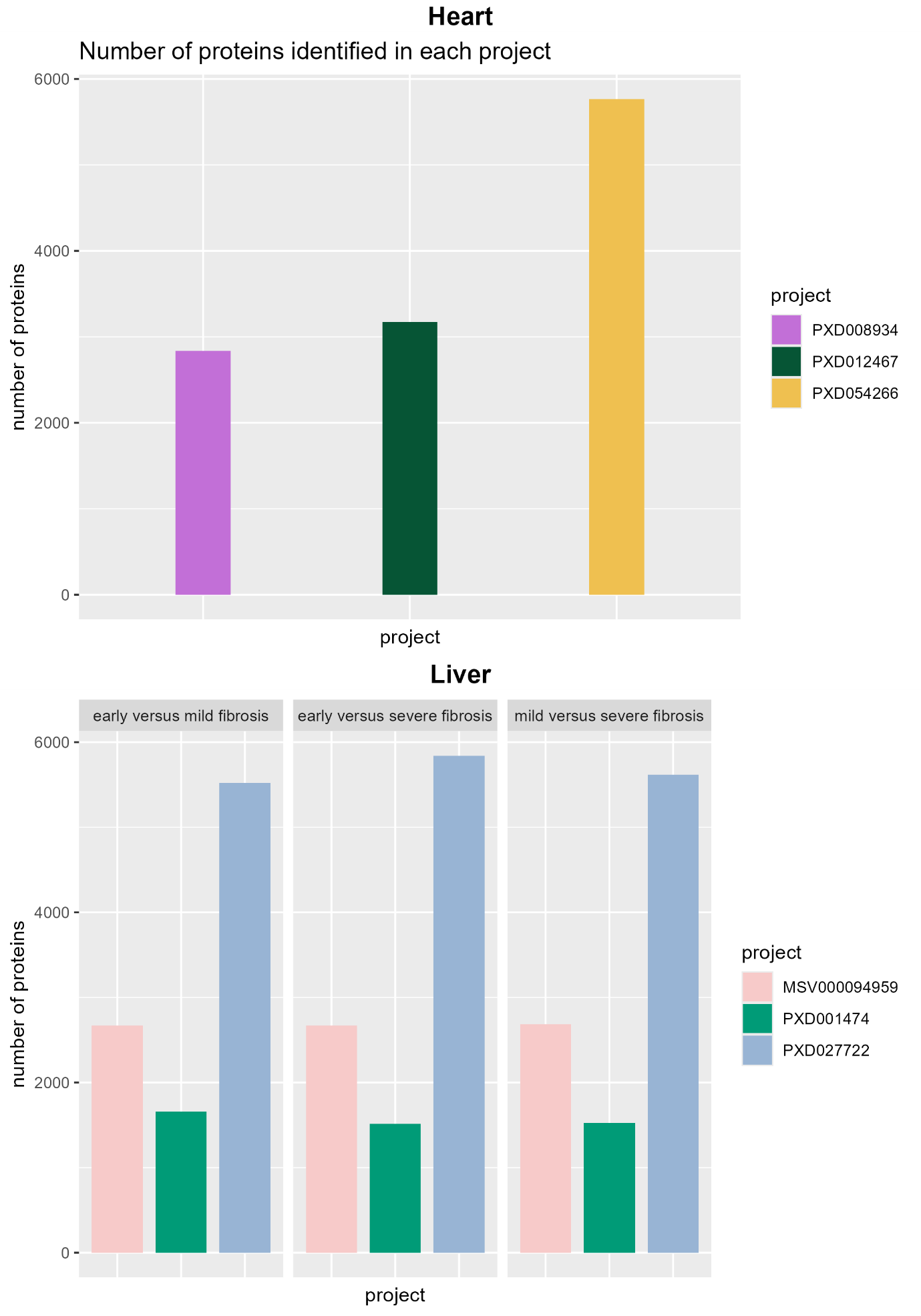
Figure S1: Overview of the number of identified proteins in each dataset, for each comparison. For heart fibrosis, fibrosis cases were compared with controls. For liver fibrosis, information on the stage of fibrosis was available, and used to compare early stage (F0) to mild stage (F1-F2), early stage (F0) to severe stage (F3-F4), and mild stage (F1-F2) to severe stage (F3-F4). Datasets considered are heart fibrosis dataset 1 (PXD008934), heart fibrosis dataset 2 (PXD012467), heart fibrosis dataset 3 (PXD054266), liver fibrosis dataset 1 (PXD001474), liver fibrosis dataset 2 (PXD027722), liver fibrosis dataset 3 (MSV000094959).

The overlap of proteins between the different datasets, after filtering as described above, is shown in **Figure S2**. In general, there is good overlap between datasets that are similar in number of identified proteins (e.g. PXD008934 and PXD012467; PXD001474 and MSV000094959, respectively).


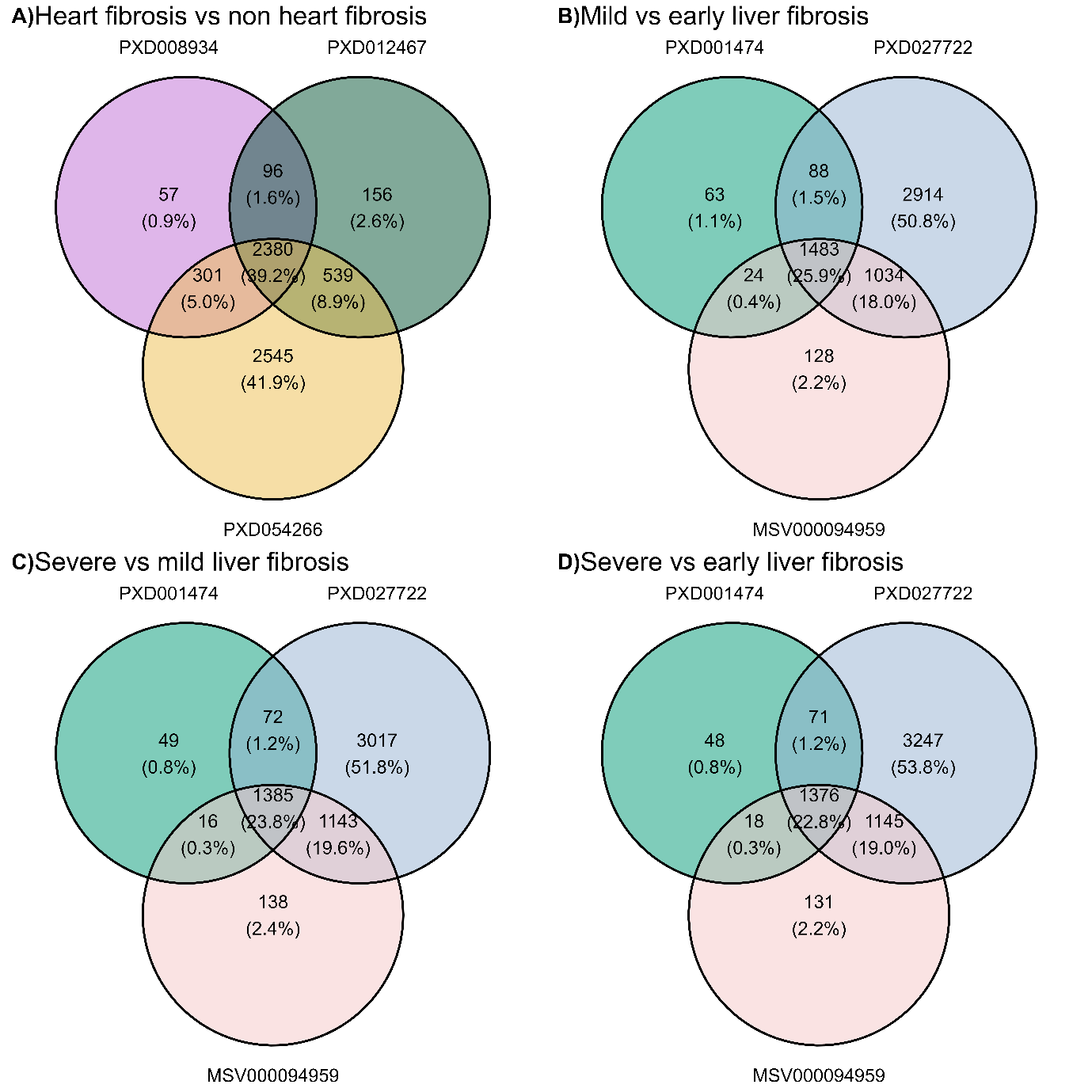


Figure S2: A) Overview of the overlap of the three datasets focusing on heart fibrosis dataset 1 (PXD008934), heart fibrosis dataset 2 (PXD012467), heart fibrosis dataset 3 (PXD054266)). Only proteins shared between at least two datasets were retained for statistical analysis. B) Overview of the overlap of the three datasets focusing on liver fibrosis, with only the early (stage F0) and mild fibrosis samples (stage F1-F2) (liver fibrosis dataset 1 (PXD001474), liver fibrosis dataset 2 (PXD027722), liver fibrosis dataset 3 (MSV000094959)). Only proteins shared between at least two datasets were retained for statistical analysis. C) Overview of the overlap of the three datasets focusing on liver fibrosis, with only the mild (stage F1-F2) and severe fibrosis samples (stage F3-F4) (liver fibrosis dataset 1 (PXD001474), liver fibrosis dataset 2 (PXD027722), liver fibrosis dataset 3 (MSV000094959)). Only proteins shared between at least two datasets were retained for statistical analysis. D) Overview of the overlap of the three datasets focusing on liver fibrosis, with only the early (stage F0) and severe fibrosis samples (stage F3-F4) (liver fibrosis dataset 1 (PXD001474), liver fibrosis dataset 2 (PXD027722), liver fibrosis dataset 3 (MSV000094959)). Only proteins shared between at least two datasets were retained for statistical analysis.

Lastly, following parts per million (ppm) normalization and log2-transformation, boxplots were generated to assess the successfulness of normalization (**Figure S3** and **4**), and Principal Component Analysis (PCA) was performed (**Figure S5** and **6**), showing distinct separation between organs and datasets.


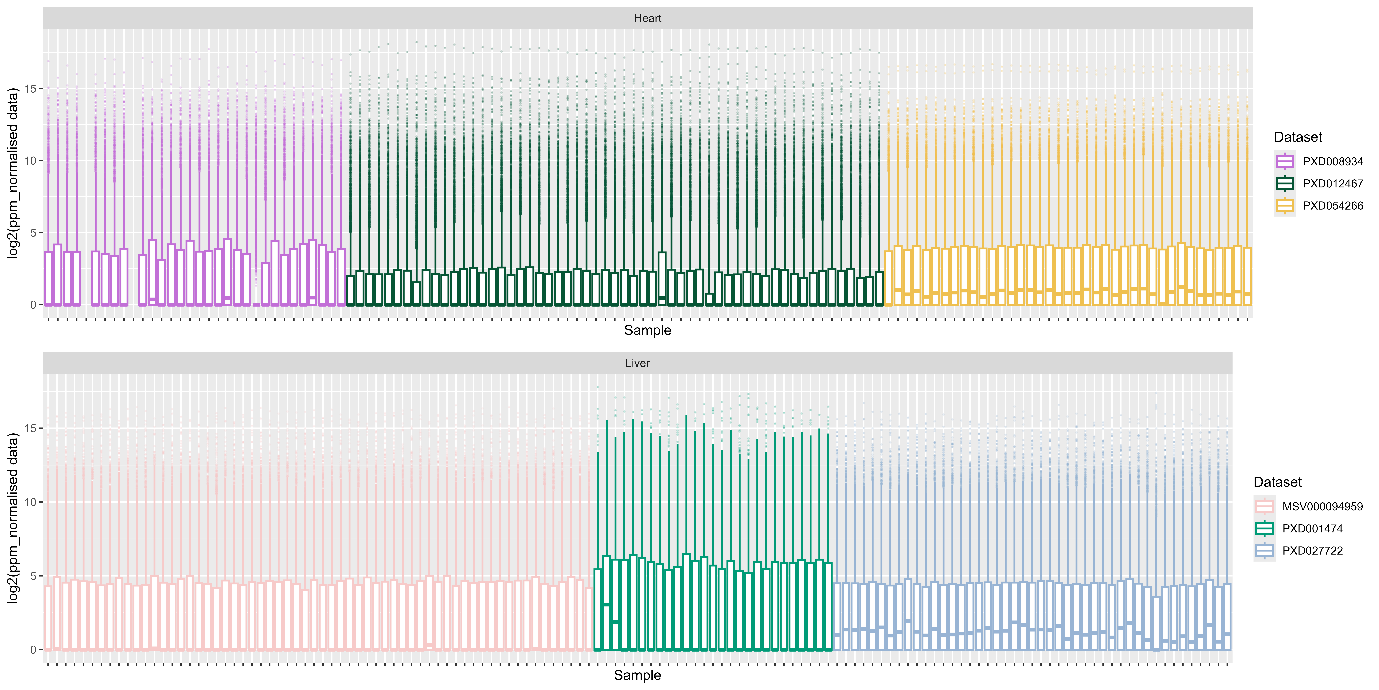
Figure S3: Boxplots of ppm-normalised, log2-transformed abundance values of each sample in each dataset in each organ. Each boxplot corresponds to one sample, and is coloured to the dataset it belongs to. Datasets considered are heart fibrosis dataset 1 (PXD008934), heart fibrosis dataset 2 (PXD012467), heart fibrosis dataset 3 (PXD054266), liver fibrosis dataset 1 (PXD001474), liver fibrosis dataset 2 (PXD027722) and liver fibrosis dataset 3 (MSV000094959).


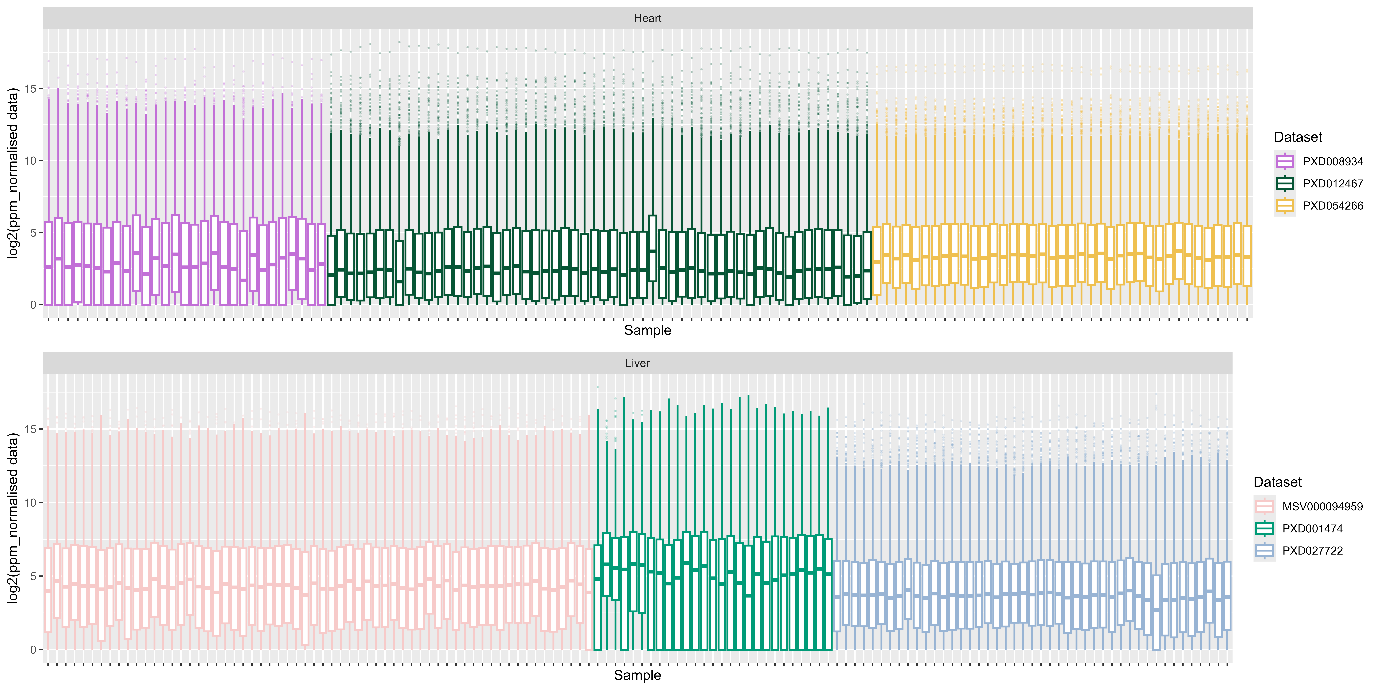
Figure S4: Boxplots following quality control of ppm-normalised, log2-transformed abundance values of each sample in each dataset in each organ. Each boxplot corresponds to one sample, and is coloured to the dataset it belongs to. Samples and proteins with more than 70% missing values were removed. Datasets considered are heart fibrosis dataset 1 (PXD008934), heart fibrosis dataset 2 (PXD012467), heart fibrosis dataset 3 (PXD054266), liver fibrosis dataset 1 (PXD001474), liver fibrosis dataset 2 (PXD027722) and liver fibrosis dataset 3 (MSV000094959).


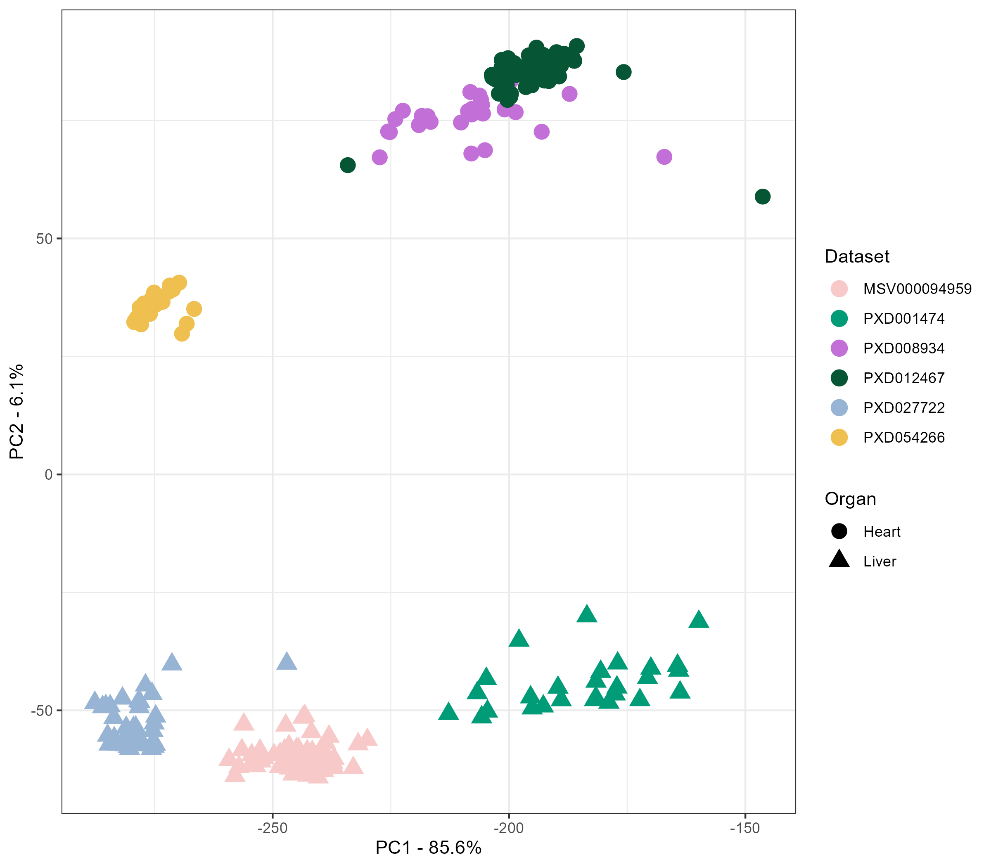


Figure S5: Principal Component Analysis (PCA) of all samples, all datasets. Samples cluster mostly within the same dataset, and then within the same organ. A slight separation of the datasets acquired using data-dependent acquisition (DDA) (heart fibrosis dataset 1 (PXD008934), heart fibrosis dataset 2 (PXD012467), liver fibrosis dataset 1 (PXD001474) and the datasets acquired using data-independent acquisition (DIA) (heart fibrosis dataset 3 (PXD054266), liver fibrosis dataset 2 (PXD027722), liver fibrosis dataset 3 (MSV000094959)) can also be observed. Percentages on the axes correspond to the explained variance of the respective component. Datasets considered are heart fibrosis dataset 1 (PXD008934), heart fibrosis dataset 2 (PXD012467), heart fibrosis dataset 3 (PXD054266), liver fibrosis dataset 1 (PXD001474), Liver fibrosis dataset 2 (PXD027722), Liver fibrosis dataset 3 (MSV000094959).


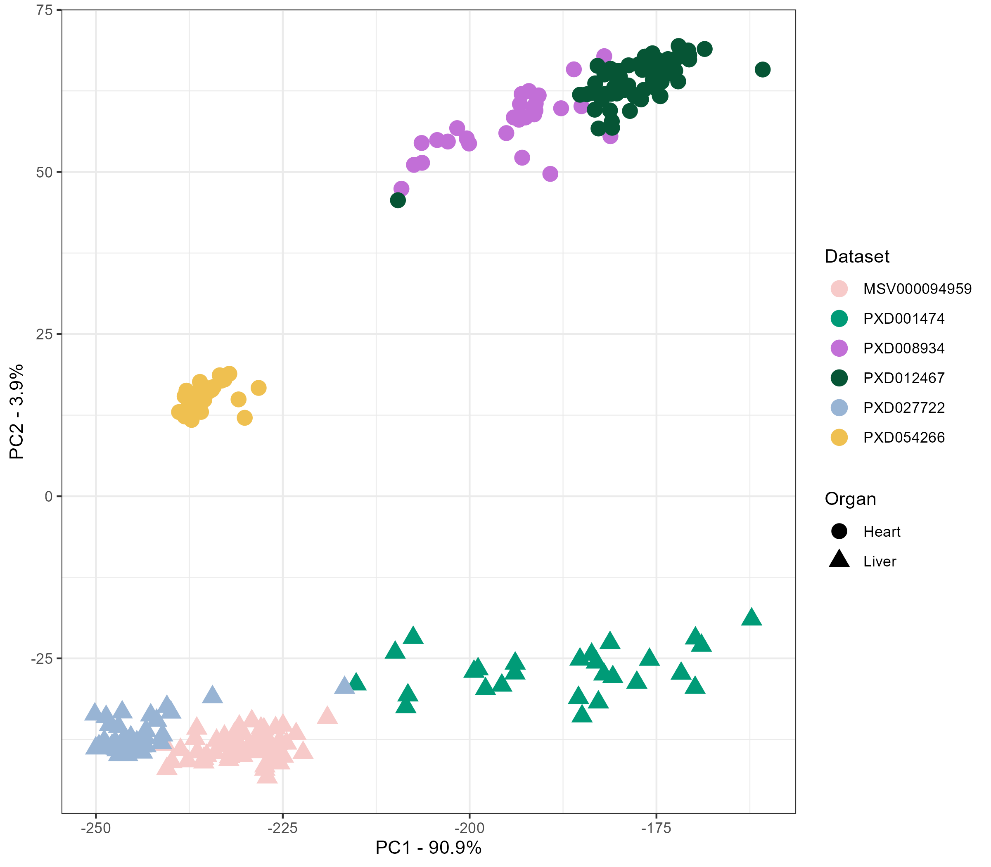


Figure S6: Principal Component Analysis (PCA) of all samples and all datasets, after applying quality control as outlined above. Samples cluster mostly within the same dataset, and then within the same organ. A slight separation of the datasets acquired using data-dependent acquisition (DDA) (heart fibrosis dataset 1 (PXD008934), heart fibrosis dataset 2 (PXD012467), liver fibrosis dataset 1 (PXD001474)) and the datasets acquired using data-independent acquisition (DIA) (heart fibrosis dataset 3 (PXD054266), liver fibrosis dataset 2 (PXD027722), liver fibrosis dataset 3 (MSV000094959)) can also be observed. Percentages on the axes correspond to the explained variance of the respective component. Datasets considered are heart fibrosis dataset 1 (PXD008934), heart fibrosis dataset 2 (PXD012467), heart fibrosis dataset 3 (PXD054266), liver fibrosis dataset 1 (PXD001474), liver fibrosis dataset 2 (PXD027722), liver fibrosis dataset 3 (MSV000094959).

Given the wide range of number of samples (from 25 in total for heart fibrosis dataset 1 (PXD008934) (heart fibrosis) to 62 for liver fibrosis dataset 3 (MSV000094959) (liver fibrosis)), as well as the uneven distribution of cases and controls especially in heart fibrosis datasets, and the considerable differences in number of identified proteins (ranging from 2822 in liver fibrosis dataset 1 (PXD001474) (liver fibrosis) until 9825 in heart fibrosis dataset 3 (PXD054266) (heart fibrosis), largely explained by the recent advancements in mass spectrometry instrumentation used in the datasets identifying more proteins as also listed in **Table S1**), and the discrepancies observed between datasets in **Figures S3-6**, statistical analysis was performed on each dataset separately and the results integrated, first on the organ level and later combining heart fibrosis and liver fibrosis, as described in more detail in the next section.

**Statistical analysis: identifying differentially expressed proteins**

After filtering out proteins with more than 70% missing values as described above, missing values were imputed using the missForest algorithm (after log_2_-transformation), based on a recent review comparing different imputation methods highlighting missForest as the best imputation method for *missing at completely random* values, and its use by the mass spectrometry community [7]. Following this, data was back-transformed, ppm normalised, log_2_-transformed, and Wilcoxon rank sum tests were carried out. For heart fibrosis, cases were compared to non-fibrotic controls, due to lack of fibrotic stage information. For liver, three comparisons were made: liver fibrosis stage 0 (early stage) versus liver fibrosis stage 1 or 2 (mild stage); liver fibrosis stage 1 or 2 (mild stage) versus liver fibrosis stage 3 or 4 (severe stage); and liver fibrosis stage 0 (early stage) versus liver fibrosis stage 3 or 4 (severe stage).

Proteins shared across at least two datasets in heart and liver were retained. All proteins for the one remaining kidney dataset were also retained. The Benjamini-Hochberg correction for multiple testing was applied to the p-values obtained from each comparison. Proteins were then further filtered to retain only proteins I) significant after Benjamini-Hochberg correction in at least one dataset; II) nominally significant in at least one dataset other than the one for which significance after correction for multiple testing was observed; III) sharing the same trend in log_2_ fold change across all datasets or across two datasets in case the protein was not retained in the third dataset. These proteins are referred to as “shared differential expressed proteins”. These proteins are listed in **Table S3** (heart fibrosis), **Table S4** (early-versus-mild liver fibrosis), **Table S5** (mild-versus-severe liver fibrosis) and **Table S6** (early-versus-severe liver fibrosis). These supplementary tables also include, for each protein, the average of the ppm normalised, log_2_-transformed abundance values of cases and controls, as well as their respective standard deviation. In addition, log_2_ fold change and p-values (corrected and uncorrected) are reported. These metrics are available for each protein and each dataset. Finally, common proteins between the shared differential expressed proteins in heart and liver were required to share the same trend in log_2_ fold change in both organs. A full list of these eighteen proteins is given below, along with their role in heart and liver fibrosis.

Table B: Overview of the eighteen shared proteins between heart and liver fibrosis, along with the results of a literature search on their role in heart and liver fibrosis, respectively.

| **Protein** | **Abbreviation in Figure 1** | **Role in heart fibrosis** | **Role in liver fibrosis** |
| --- | --- | --- | --- |
| **Collagen type V alpha 1** | COLα1 (V) | limits fibrotic scar size [8] | Limits the growth of collagen type I fibrils in fibrotic liver [9] |
| **Collagen type V alpha 2** | COLα2 (V) |  |  |
| **Collagen type VI alpha 2** | COLα2 (VI) | Forms a network with fibrillar collagens [10] | Has affinity for pro-MMP-1, -8, -13 and MMP-3, aiding in their activation [11] |
| **Collagen type XIV alpha 1** | COLα1 (XIV) | Moderates scar formation [12] | Increased [13], but its function remains unclear |
| **Lumican** | LUM | Increases collagen fibril length and thickness [14,15] | |
| **Tenascin-X** | TNXB | Increases collagen fibrillogenesis [16,17] | |
| **Fibulin 5** | FBLN5 | Involved in elastic fibre formation [18,19] | |
| **Clusterin** | CLU | Reduced cardiomyocyte mortality [20] | Increases in hepatocytes, leads to hepatic stellate cell activation [21] |
| **Heat shock protein beta 6** | HSPB6 | Increased in cardiomyocytes, correlating with cardiac dysfunction [22] | Increases in hepatocytes, signifying lipid accumulation [23], interleukin-6 secretion leading to fibroblast activation [24] |
| **Asporin** | ASPN | Upregulated in activated fibroblasts [24,25] | |
| **EGF-containing fibulin-like extracellular matrix protein 1** | EFEMP1 | Upregulated in activated fibroblasts [26,27] | |
| **Adipocyte enhancer-binding protein 1** | AEBP1 | Upregulated in activated fibroblasts [28,29] | |
| **Myosin 10** | MYH10 | Upregulated in activated fibroblasts [30,31] | |
| **Microfibril-associated glycoprotein 4** | MFAP4 | Activates integrin receptors, leading to increased Transforming Growth Factor β secretion [32], which induces MFAP4 secretion [33] | Increased in activated hepatic stellate cells [34] |
| **Immunoglobulin heavy constant gamma 1** | IGHG1 | Increased in myocardial infarction with unclear role [35] | Increased in hepatitis B-virus induced cirrhosis with unclear role [36] |
| **Transforming growth factor beta-induced protein ig-h3** | TGFBI | Binds integrins, increasing collagen synthesis [37] | |
| **Protein AMBP** | Not displayed | No direct link found | |
| **Prolargin** |  |  |  |

**StringDB analysis**

String database [38,39] was used to map interacting proteins for the shared differential expressed proteins listed in **Table S3-6**, using all different types of evidence for human proteins. Interaction partners had to have a confidence score of at least 0.7 (on a scale between 0 and 1), as listed in StringDB. So called “Singletons”, proteins for which no interaction partners could be identified, were removed from the images of the networks for increased clarity; and proteins were coloured according to upregulation (red) or downregulation (blue) across all datasets. Thickness of the edges (lines) between the nodes (proteins) corresponds to the strength of the evidence as calculated by StringDB; distance between the nodes (proteins) indicates how closely related they are. Considering only four proteins were identified as shared differential expressed proteins in early-versus-mild liver fibrosis (**Table S4**), a protein interaction network could not be generated. The remaining networks are shown in **Figures S7‑9**.


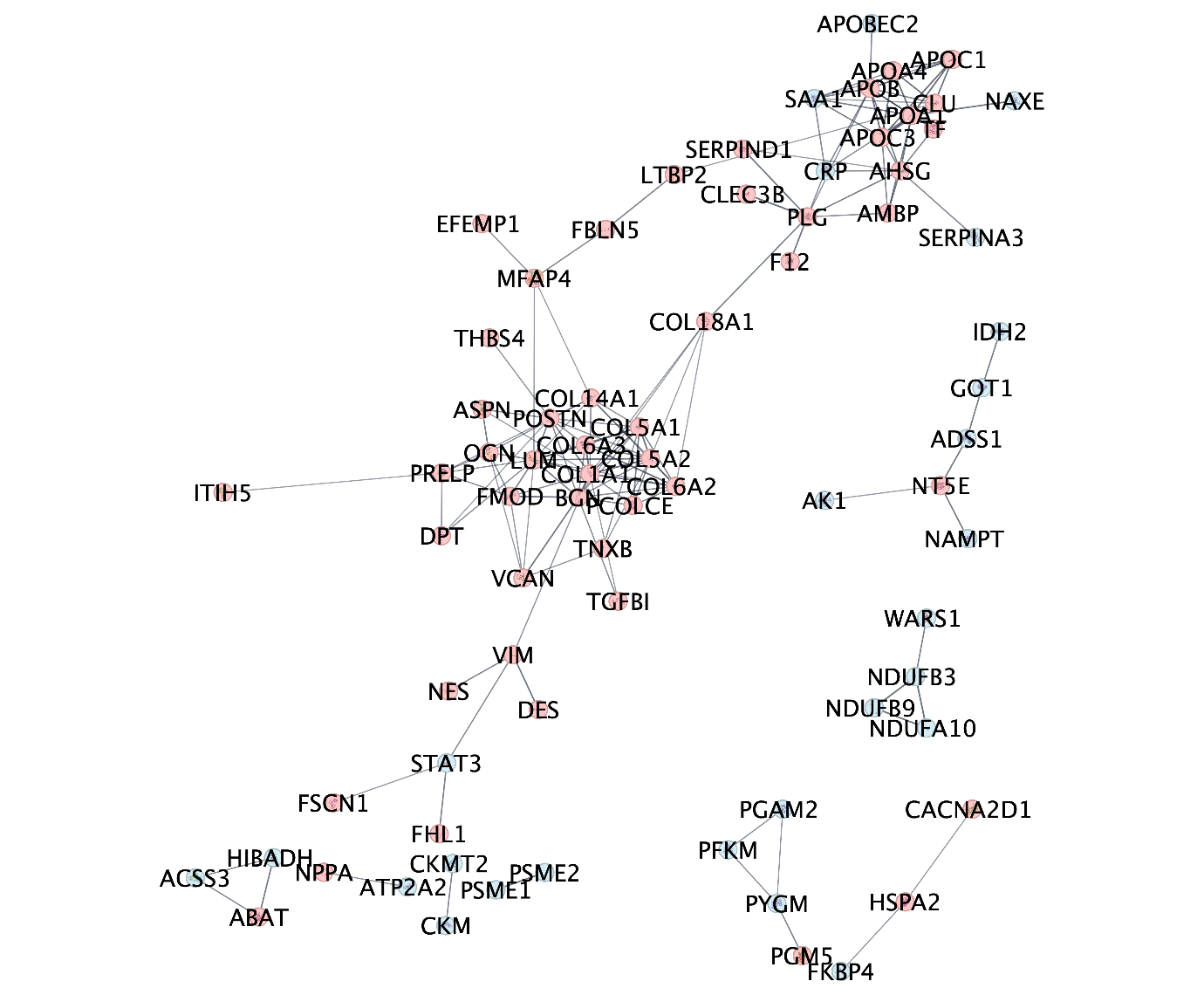


Figure S7: StringDB [38,39] interaction network of the 124 shared differential expressed proteins in heart fibrosis, coloured according to the trend in log_2_ fold change (red: upregulated; blue: downregulated) across the three datasets focussing on heart fibrosis. Thickness of the edges (lines) between the nodes (proteins) corresponds to the strength of the evidence as calculated by StringDB; distance between the nodes (proteins) indicates how closely related they are.


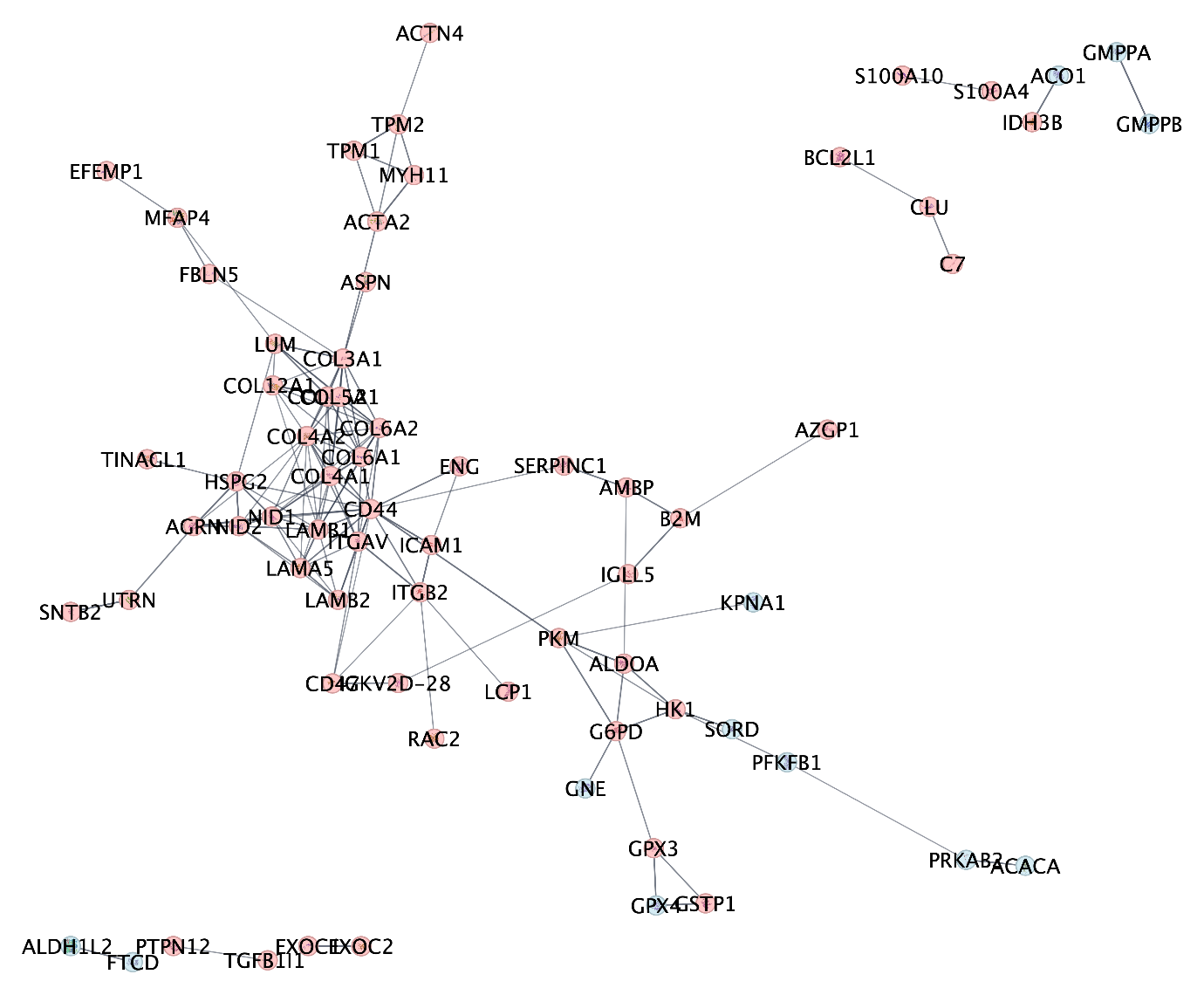


Figure S8: StringDB [38,39] interaction network of the 135 shared differential expressed proteins in mild-versus-severe liver fibrosis, coloured according to the trend in log_2_ fold change (red: upregulated; blue: downregulated) across the three datasets focussing on liver fibrosis. Thickness of the edges (lines) between the nodes (proteins) corresponds to the strength of the evidence as calculated by StringDB; distance between the nodes (proteins) indicates how closely related they are.


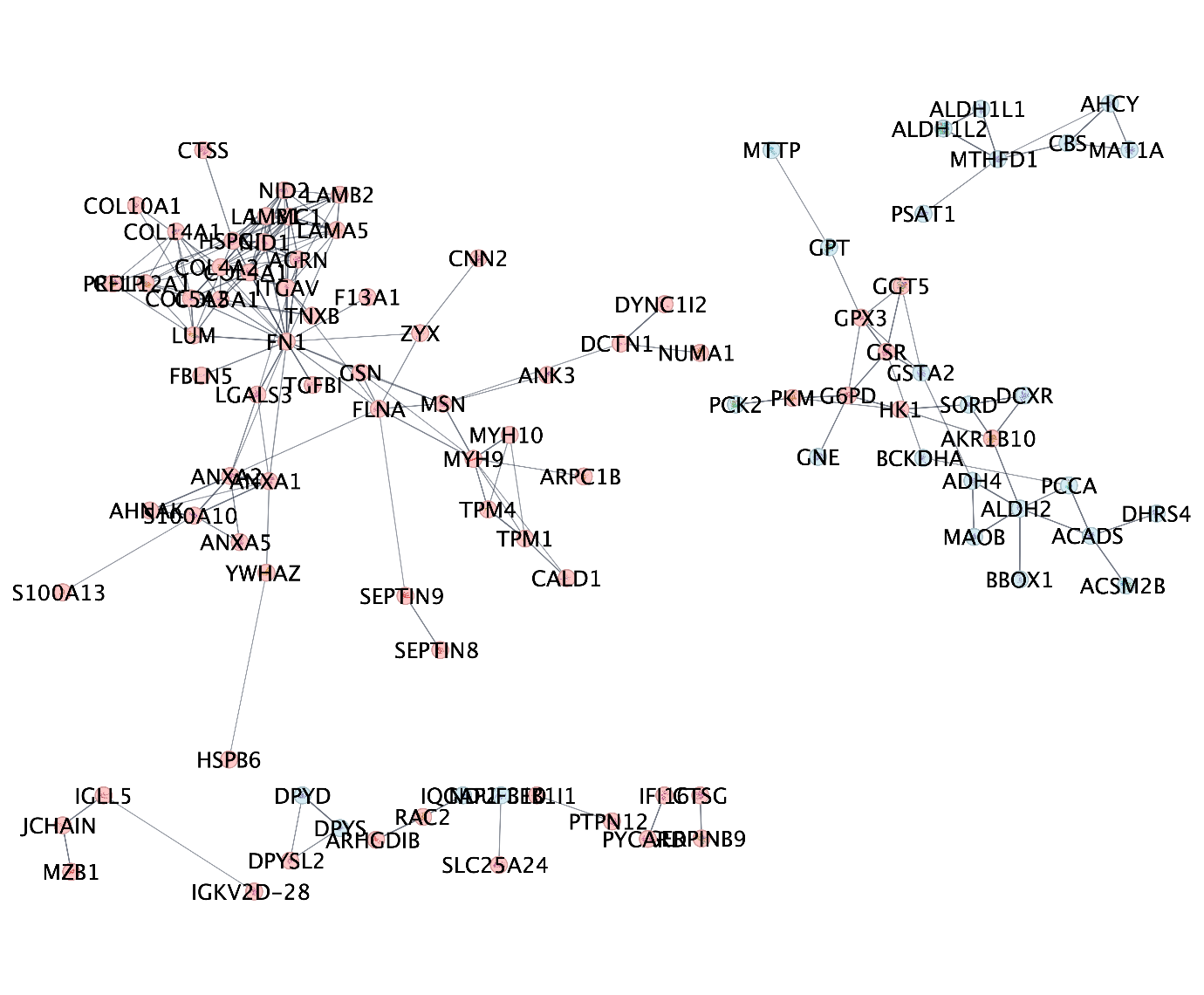


Figure S9: StringDB [38,39] interaction network of the 160 shared differential expressed proteins in early-versus-severe liver fibrosis, coloured according to the trend in log_2_ fold change (red: upregulated; blue: downregulated) across the three datasets focussing on liver fibrosis. Thickness of the edges (lines) between the nodes (proteins) corresponds to the strength of the evidence as calculated by StringDB; distance between the nodes (proteins) indicates how closely related they are.

For each network, Markov Clustering [39] was performed under the default parameters. Five clusters were identified in the heart fibrosis network, three in the mild-versus-severe liver fibrosis network and four in the early-versus-severe liver fibrosis network. Among these, one cluster from each of the networks, as well as two clusters from the heart fibrosis network and the early-versus-severe liver fibrosis network share a common biological function and similar changes in log_2_ fold change (**Figure S10-11**).


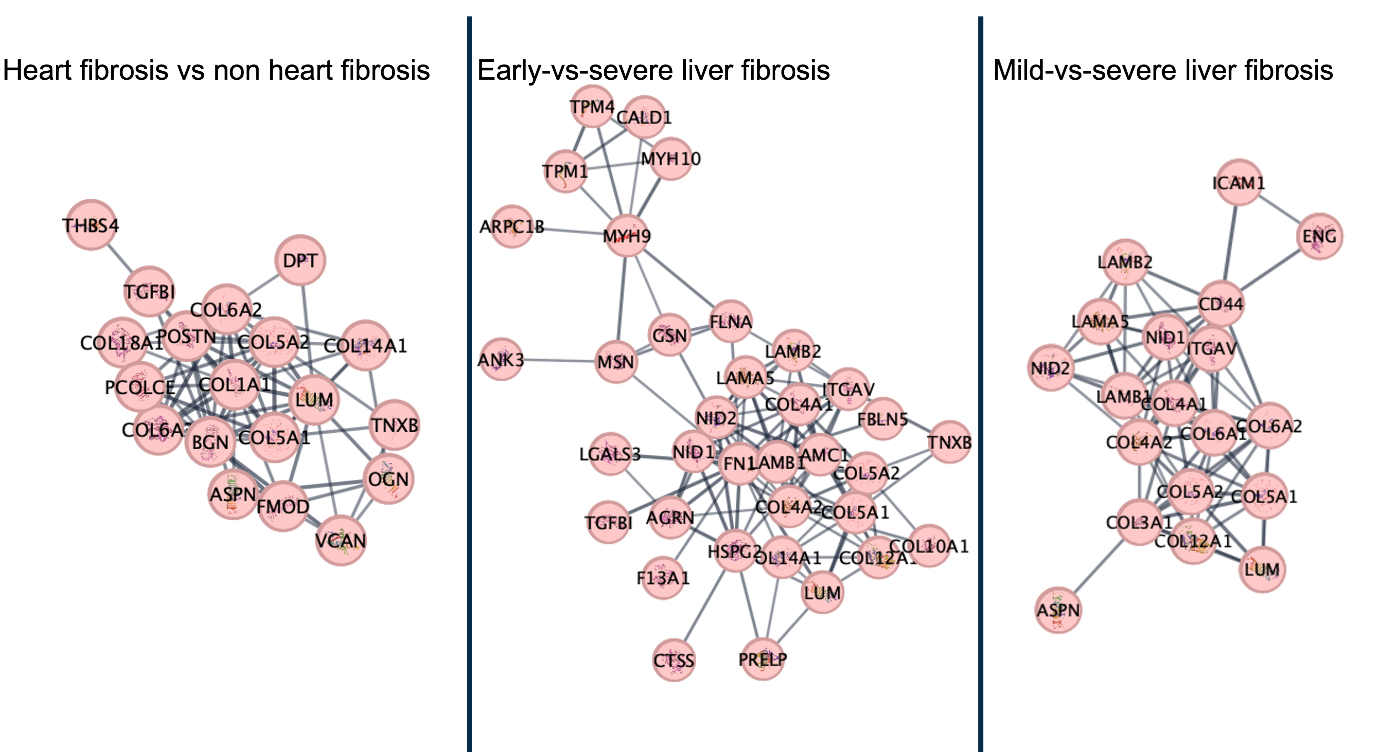


Figure S10: Cluster of ECM proteins, all upregulated (as indicated by the red colour), identified in heart fibrosis, early-versus-severe liver fibrosis and mild-versus-severe liver fibrosis (from left to right).


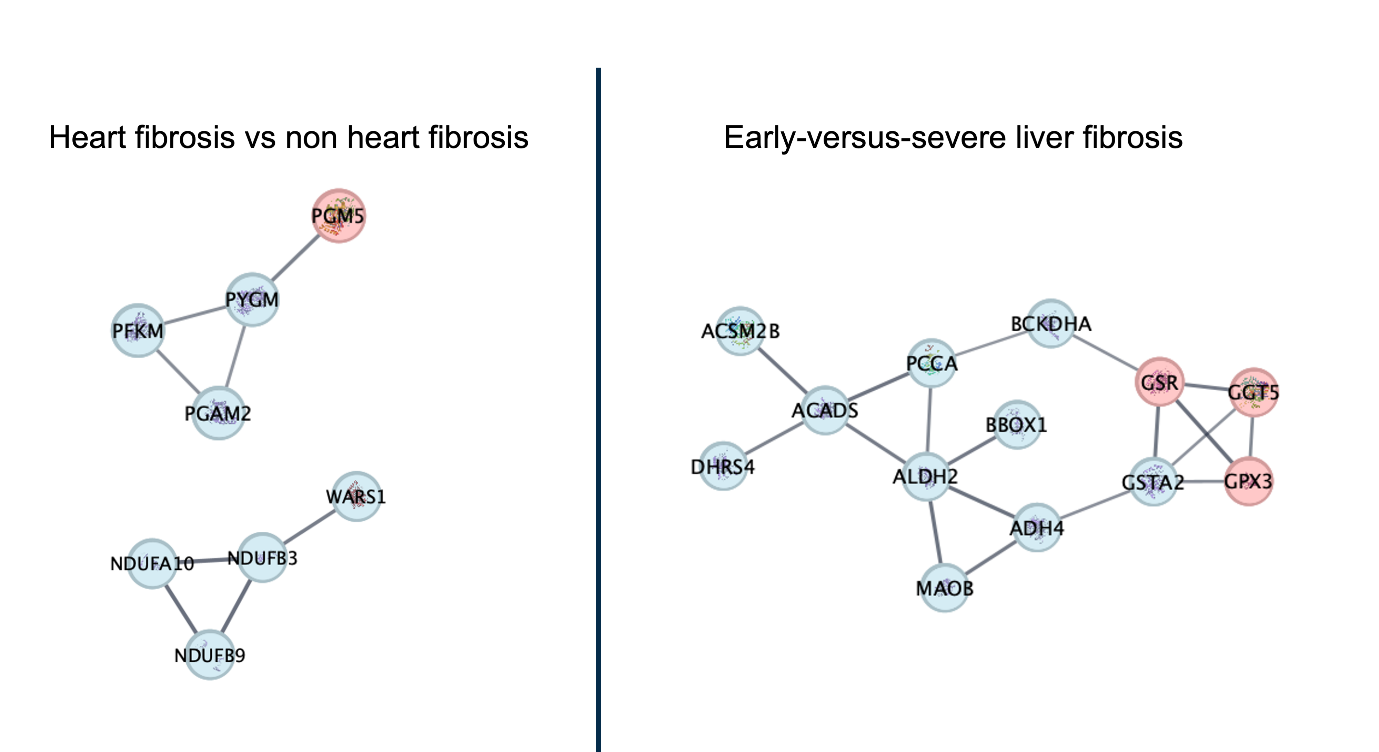


Figure S11: Cluster of proteins associated with cell energy metabolism and mitochondrial activity, mostly downregulated (downregulated: blue colour, upregulated: red colour), identified in heart fibrosis and early-versus-severe liver fibrosis (from left to right).

**Gene Set Variation Analysis (GSVA)**

Annotation from the Molecular Signature Database MSigDB [40] was obtained; specifically, gene sets from reactome, and gene ontology (biological pathways, molecular function, cellular component) and hallmark gene sets. For GSVA [41,42], only gene sets containing at least five genes that were also present in the proteomics dataset analysed, were retained; and enrichment scores were normalized by dividing by the absolute difference between the minimum and maximum enrichment score of that gene set. As input, genes corresponding to the proteins listed as shared differential expressed proteins in heart fibrosis (n=124), shared differential expressed proteins in mild-versus-severe liver fibrosis (n=135), and shared differential expressed proteins in early-versus-severe liver fibrosis were used (n=160), for each dataset separately. Gene sets were then further filtered by selecting gene sets demonstrating significant changes in enrichment scores (after Benjamini-Hochberg correction) in at least two datasets per organ, and sharing the same trend in log_2_ fold change in enrichment score across all datasets of the same organ (e.g. (de-)activated in all three datasets focusing on heart fibrosis), and retained in at least two out of three datasets. Shared differentially (de-)activated pathways in both heart and liver fibrosis were then selected. Pathways were manually inspected to remove duplicate pathways, only retaining the pathway with the highest coverage. The fifteen most significant (de-)activated pathways shared between heart fibrosis and mild-versus-severe liver fibrosis, and heart fibrosis and early-versus-severe liver fibrosis are shown in **Figure S12, panels A-D**, and the full list is available in **Table S8-9**, respectively.


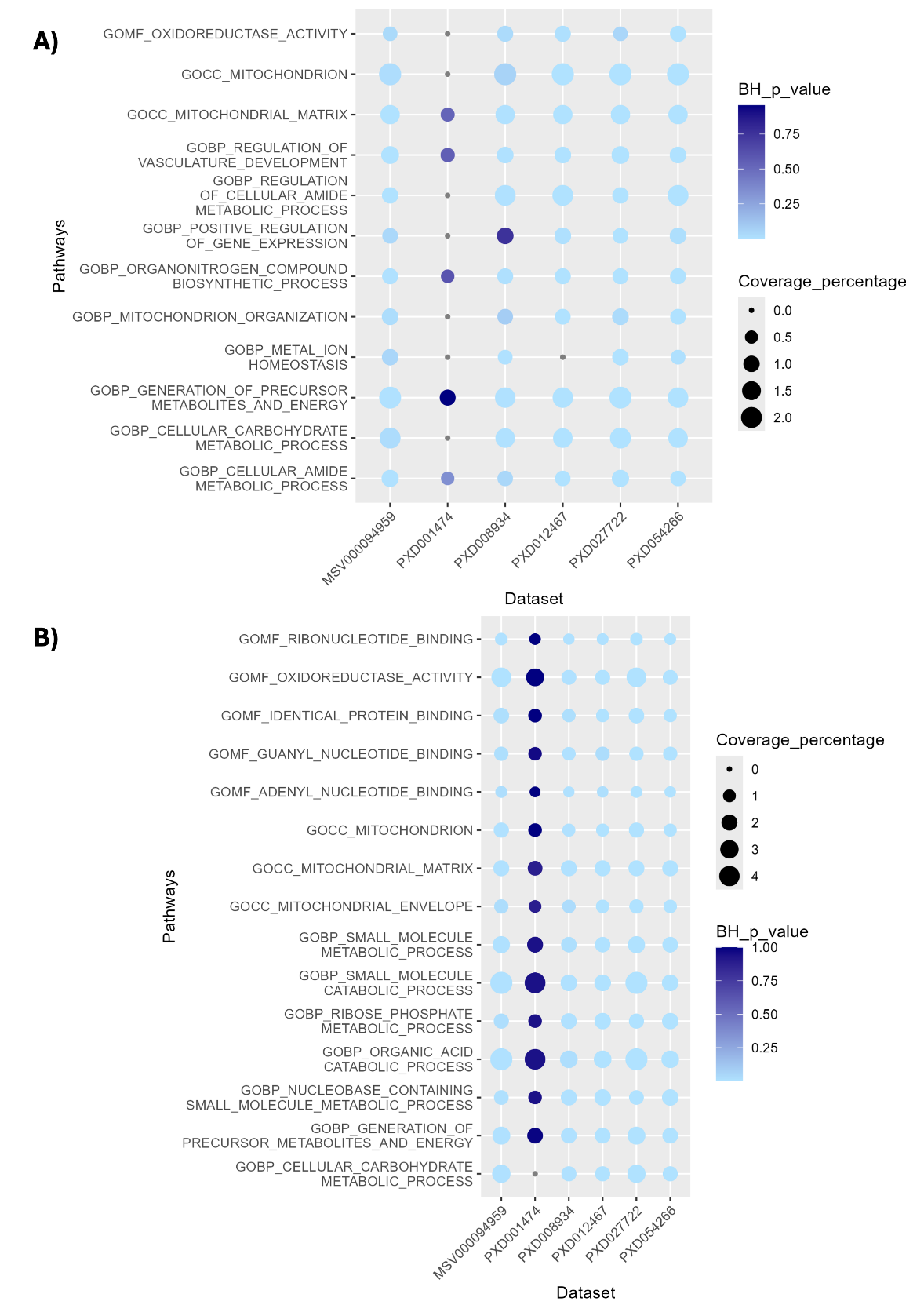


Figure S12: Overview of the top 15 most significantly deactivated pathways. Colour indicates the Benjamini-Hochberg (BH)-corrected p-value in the respective dataset; size of the dot indicates the percentage proteins identified in our data over the total number of proteins in that gene set. Panel A) shows the common deactivated pathways for heart fibrosis and mild-versus-severe liver fibrosis: panel B) shows the same for heart fibrosis and early-versus-severe liver fibrosis. Datasets considered are heart fibrosis dataset 1 (PXD008934), heart fibrosis dataset 2 (PXD012467), heart fibrosis dataset 3 (PXD054266), liver fibrosis dataset 1 (PXD001474), liver fibrosis dataset 2 (PXD027722) and liver fibrosis dataset 3 (MSV000094959).


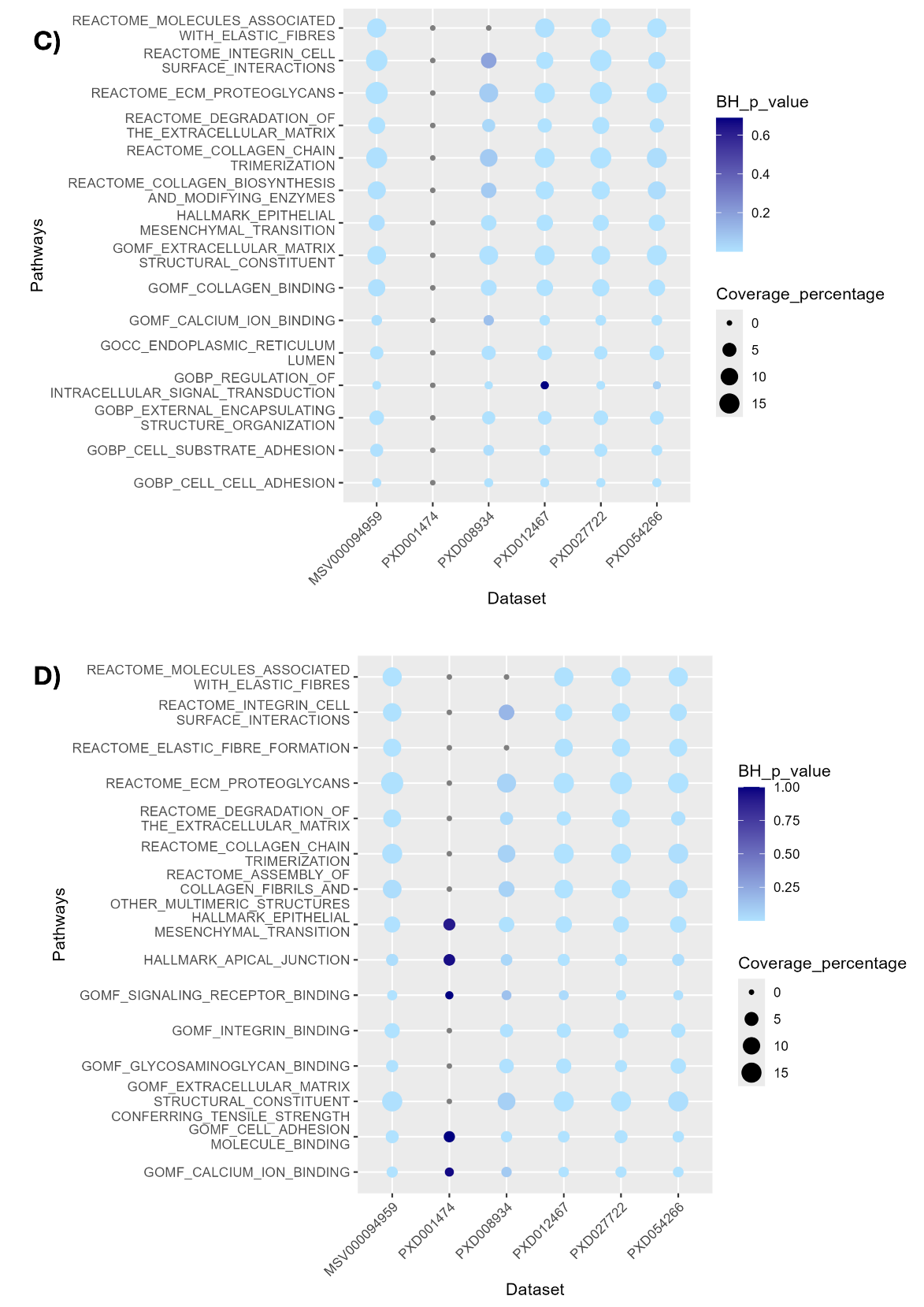


Figure S12 continued: Overview of the top 15 most significantly activated pathways. Colour indicates the Benjamini-Hochberg (BH)-corrected p-value in the respective dataset; size of the dot indicates the percentage proteins identified in our data over the total number of proteins in that gene set. Panel C) shows the common activated pathways for heart fibrosis and mild-versus-severe liver fibrosis: panel D) shows the same for heart fibrosis and early-versus-severe liver fibrosis. Datasets considered are heart fibrosis dataset 1 (PXD008934), heart fibrosis dataset 2 (PXD012467), heart fibrosis dataset 3 (PXD054266), liver fibrosis dataset 1 (PXD001474), liver fibrosis dataset 2 (PXD027722) and liver fibrosis dataset 3 (MSV000094959).

**Network-based Drug Repurposing and Exploration (NedRex)**

For Network-based Drug Repurposing and exploration (NedRex [43]), we employed Cytoscape version 3.10.3 [44], using the shared proteins in heart fibrosis, the shared proteins in early-versus-mild liver fibrosis, and the shared proteins in early-versus-late liver fibrosis, respectively. UniProt Protein IDs were mapped to Entrez gene ids using UniProtKB’s ID mapping tool (https://www.uniprot.org/id-mapping). Two confidence ranking algorithms were used: the TrustRank algorithm and the Closeness Centrality algorithm. The following parameters were applied in both algorithms: result size is 100, include direct and indirect drugs, included approved drugs and drug which are not approved. For the TrustRank algorithm, the damping factor was specified at 0.85. Finally, these results were integrated into one candidate fibrotic drug list, retaining only drug candidates identified for heart fibrosis and either of the two comparisons of liver fibrosis, by either the TrustRank or the Closeness Centrality algorithm (**Table S10**).

**References**

[1] Stroggilos, R., Tserga, A., Zoidakis, J., Vlahou, A., Makridakis, M., Tissue proteomics repositories for data reanalysis. *Mass Spectrom. Rev.* 2024, 43, 1270–1284.

[2] Loizos, S., Shiakalli Chrysa, T., Christos, G.S., Amyloidosis: Review and Imaging Findings. *Semin. Ultrasound CT MRI* 2014, 35, 225–239.

[3] Wynn, T.A., Ramalingam, T.R., Mechanisms of fibrosis: therapeutic translation for fibrotic disease. *Nat. Med.* 2012, 18, 1028–1040.

[4] Tandon, P., Abrams, N.D., Avula, L.R., Carrick, D.M., et al., Unraveling Links between Chronic Inflammation and Long COVID: Workshop Report. *J. Immunol. Baltim. Md 1950* 2024, 212, 505–512.

[5] Jiao, J., Ji, L., Li, X., Gao, Z., et al., Dynamic proteomic change of tumor and immune organs in an immune-competent hepatocellular carcinoma mouse model. *Am. J. Cancer Res.* 2022, 12, 1621–1634.

[6] Dzeshka, M.S., Lip, G.Y.H., Snezhitskiy, V., Shantsila, E., Cardiac Fibrosis in Patients With Atrial Fibrillation: Mechanisms and Clinical Implications. *J. Am. Coll. Cardiol.* 2015, 66, 943–959.

[7] Schumann, Y., Gocke, A., Neumann, J.E., Computational Methods for Data Integration and Imputation of Missing Values in Omics Datasets. *PROTEOMICS* 2024, 25, e202400100.

[8] Yokota, T., McCourt, J., Ma, F., Ren, S., et al., Type V Collagen in Scar Tissue Regulates the Size of Scar after Heart Injury. *Cell* 2020, 182, 545-562.e23.

[9] Mak, K.M., Png, C.Y.M., Lee, D.J., Type V Collagen in Health, Disease, and Fibrosis. *Anat. Rec.* 2016, 299, 613–629.

[10] Li, L., Zhao, Q., Kong, W., Extracellular matrix remodeling and cardiac fibrosis. *Matrix Biol.* 2018, 68–69, 490–506.

[11] Freise, C., Erben, U., Muche, M., Farndale, R., et al., The alpha 2 chain of collagen type VI sequesters latent proforms of matrix-metalloproteinases and modulates their activation and activity. *Matrix Biol.* 2009, 28, 480–489.

[12] Tao, G., Levay, A.K., Peacock, J.D., Huk, D.J., et al., Collagen XIV is important for growth and structural integrity of the myocardium. *J. Mol. Cell. Cardiol.* 2012, 53, 626–638.

[13] Collagen biology and non‐invasive biomarkers of liver fibrosis - Karsdal - 2020 - Liver International - Wiley Online Library n.d.

[14] Rixon, C., Andreassen, K., Shen, X., Erusappan, P.M., et al., Lumican accumulates with fibrillar collagen in fibrosis in hypertrophic cardiomyopathy. *ESC Heart Fail.* 2023, 10, 858–871.

[15] Charlton, M., Viker, K., Krishnan, A., Sanderson, S., et al., Differential expression of lumican and fatty acid binding protein-1: New insights into the histologic spectrum of nonalcoholic fatty liver disease. *Hepatology* 2009, 49, 1375–1384.

[16] Jing, L., Zhou, L.-J., Zhang, F.-M., Li, W.-M., Sang, Y., Tenascin-x facilitates myocardial fibrosis and cardiac remodeling through transforming growth factor-β1 and peroxisome proliferator-activated receptor γ in alcoholic cardiomyopathy. *Chin. Med. J. (Engl.)* 2011, 124, 390–395.

[17] Yamaguchi, S., Kawakami, K., Satoh, K., Fukunaga, N., et al., Suppression of hepatic dysfunction in tenascin-X-deficient mice fed a high-fat diet. *Mol. Med. Rep.* 2017, 16, 4061–4067.

[18] Běhounek, M., Lipcseyová, D., Vít, O., Žáček, P., et al., Biomarkers of RV Dysfunction in HFrEF Identified by Direct Tissue Proteomics: Extracellular Proteins Fibromodulin and Fibulin-5. *Circ. Heart Fail.* 2025, 18, e011984.

[19] Pantano, L., Agyapong, G., Shen, Y., Zhuo, Z., et al., Molecular characterization and cell type composition deconvolution of fibrosis in NAFLD. *Sci. Rep.* 2021, 11, 18045.

[20] Turkieh, A., Weber, L., Chwastyniak, M., Baydar, S., et al., Regulation of Clusterin in the Heart and Plasma of Mice After Transverse Aortic Constriction. *J. Cell. Mol. Med.* 2024, 28, e70290.

[21] Seo, H.-Y., Lee, S.-H., Lee, J.-H., Kang, Y.N., et al., Clusterin Attenuates Hepatic Fibrosis by Inhibiting Hepatic Stellate Cell Activation and Downregulating the Smad3 Signaling Pathway. *Cells* 2019, 8, 1442.

[22] Turkieh, A., Weber, L., Chwastyniak, M., Baydar, S., et al., Regulation of Clusterin in the Heart and Plasma of Mice After Transverse Aortic Constriction. *J. Cell. Mol. Med.* 2024, 28, e70290.

[23] Miao, Y., Zhong, Y., Li, Y., Qin, H., et al., Inhibition of HSP20 Ameliorates Steatotic Liver Disease by Stimulating ERK2-Dependent Autophagy. *Diabetes* 2024, 73, 909–925.

[24] Major, J.L., McKinsey, T.A., Putting the Heat on Cardiac Fibrosis. *JACC Basic Transl. Sci.* 2019, 4, 200–203.

[25] Kumagai, H., Sasaki, A., Umemura, A., Kakisaka, K., et al., Effects of laparoscopic sleeve gastrectomy on nonalcoholic fatty liver disease and TGF-β signaling pathway. *Endocr. J.* 2024, 71, 139–152.

[26] Murtha, L.A., Hardy, S.A., Mabotuwana, N.S., Bigland, M.J., et al., Fibulin-3 is necessary to prevent cardiac rupture following myocardial infarction. *Sci. Rep.* 2023, 13, 14995.

[27] Kim, H.Y., Rosenthal, S.B., Liu, X., Miciano, C., et al., Multi-modal analysis of human hepatic stellate cells identifies novel therapeutic targets for metabolic dysfunction-associated steatotic liver disease. *J. Hepatol.* 2024.

[28] Rao, M., Wang, X., Guo, G., Wang, L., et al., Resolving the intertwining of inflammation and fibrosis in human heart failure at single-cell level. *Basic Res. Cardiol.* 2021, 116, 55.

[29] Zhang, W., Li, Y.J., Zhang, N., Chen, S.Y., et al., Fibroblast-specific adipocyte enhancer binding protein 1 is a potential pathological trigger and prognostic marker for liver fibrosis independent of etiology. *J. Dig. Dis.* 2023, 24, 550–561.

[30] Fan, H., Tan, X., Xu, S., Zeng, Y., et al., Identification and validation of differentially expressed disulfidptosis-related genes in hypertrophic cardiomyopathy. *Mol. Med.* 2024, 30, 249.

[31] Kim, H.-T., Yin, W., Jin, Y.-J., Panza, P., et al., Myh10 deficiency leads to defective extracellular matrix remodeling and pulmonary disease. *Nat. Commun.* 2018, 9, 4600.

[32] Wang, H., Yang, J., Shuai, W., Yang, J., et al., Deletion of Microfibrillar‐Associated Protein 4 Attenuates Left Ventricular Remodeling and Dysfunction in Heart Failure. *J. Am. Heart Assoc.* 2020, 9, e015307.

[33] Kanaan, R., Medlej-Hashim, M., Jounblat, R., Pilecki, B., Sorensen, G.L., Microfibrillar-associated protein 4 in health and disease. *Matrix Biol.* 2022, 111, 1–25.

[34] Pan, L.-X., Tian, W., Huang, Z.-H., Li, J.-R., et al., Identification of a liver fibrosis and disease progression-related transcriptome signature in non-alcoholic fatty liver disease. *Int. J. Biochem. Cell Biol.* 2025, 180, 106751.

[35] de Winter, N., Ji, J., Sintou, A., Forte, E., et al., Persistent transcriptional changes in cardiac adaptive immune cells following myocardial infarction: New evidence from the re-analysis of publicly available single cell and nuclei RNA-sequencing data sets. *J. Mol. Cell. Cardiol.* 2024, 192, 48–64.

[36] Zhang, P., Li, H., Peng, B., Zhang, Y., et al., Single-cell RNA transcriptomics reveals differences in the immune status of alcoholic and hepatitis B virus-related liver cirrhosis. *Front. Endocrinol.* 2023, 14.

[37] Altieri, A., Visser, G.V., Buechler, M.B., Enter the Matrix: Fibroblast-immune cell interactions shape extracellular matrix deposition in health and disease. *F1000Research* 2024, 13, 119.

[38] Szklarczyk, D., Kirsch, R., Koutrouli, M., Nastou, K., et al., The STRING database in 2023: protein–protein association networks and functional enrichment analyses for any sequenced genome of interest. *Nucleic Acids Res.* 2022, 51, D638–D646.

[39] Doncheva, N.T., Morris, J.H., Gorodkin, J., Jensen, L.J., Cytoscape StringApp: Network Analysis and Visualization of Proteomics Data. *J. Proteome Res.* 2019, 18, 623–632.

[40] Liberzon, A., Birger, C., Thorvaldsdóttir, H., Ghandi, M., et al., The Molecular Signatures Database (MSigDB) hallmark gene set collection. *Cell Syst.* 2015, 1, 417–425.

[41] Hänzelmann, S., Castelo, R., Guinney, J., GSVA: gene set variation analysis for microarray and RNA-Seq data. *BMC Bioinformatics* 2013, 14, 7.

[42] Barbie, D.A., Tamayo, P., Boehm, J.S., Kim, S.Y., et al., Systematic RNA interference reveals that oncogenic KRAS-driven cancers require TBK1. *Nature* 2009, 462, 108–112.

[43] Sadegh, S., Skelton, J., Anastasi, E., Bernett, J., et al., Network medicine for disease module identification and drug repurposing with the NeDRex platform. *Nat. Commun.* 2021, 12, 6848.

[44] Shannon, P., Markiel, A., Ozier, O., Baliga, N.S., et al., Cytoscape: a software environment for integrated models of biomolecular interaction networks. *Genome Res.* 2003, 13, 2498–2504.
